# Supplementary material for: The circadian calling activity of a lebinthine cricket with high-frequency calls is unaffected by cicada choruses in the day
Source: PeerJ. 2023 Jan 12;11:e14641. doi: 10.7717/peerj.14641 (PMC9840852; doi:10.7717/peerj.14641)
Supplement: Supplemental Information 2 — +1 refers to the next following day. [file peerj-11-14641-s002.pdf]

Table S1. Description of the calling activity and peak frequency of some sympatric low-frequency calling crickets and katydids. $^{+1}$ refers to the next following day.

**Species**	**Calling activity**	**Peak frequency (kHz)**	**Reference**
Ornebius spp.	21h–4h^{+1}	6.1–8.1	Tan et al. under review
Gymnogryllus spp.	18h–4h^{+1}	4.4–5.9	Tan et al. 2018
Velarifictorus aspersus	19h–6h^{+1}<br>One case 17h–8h^{+1}	4.8–5.2	Ingrisch 1998
Mecopoda sismondoi	19h–3h^{+1}	5–10	Heller et al. 2021
